# Molecular Predictors for Advanced Papillary Thyroid Carcinoma Recurrence

**DOI:** 10.3389/fendo.2019.00839

**Published:** 2019-12-05

**Authors:** Taciana Padilha de Castro, Ricardo Cortez Cardoso Penha, Luisa Aguirre Buexm, Flávia Nascimento de Carvalho, Raquel de Vasconcellos Carvalhaes Oliveira, Fernando Vaz Agarez, Luciana Wernersbach Pinto, Denise P. Carvalho

**Affiliations:** ^1^Research Center for Health Work and Human Ecology (CESTEH-ENSP), FIOCRUZ, Rio de Janeiro, Brazil; ^2^Molecular Carcinogenesis Program, Research Center, National Institute of Cancer, Rio de Janeiro, Brazil; ^3^Clinical Epidemiology Laboratory, Evandro Chagas National Institute of Infectious Diseases, FIOCRUZ, Rio de Janeiro, Brazil; ^4^Pathology Division, National Cancer Institute, Rio de Janeiro, Brazil; ^5^Endocrine Physiology Laboratory Doris Rosenthal, Institute of Biophysics Carlos Chagas Filho, Federal University of Rio de Janeiro, Rio de Janeiro, Brazil

**Keywords:** papillary thyroid carcinoma, recurrence, BRAFV600E, progesterone receptor, E-cadherin

## Abstract

Despite its indolent course, one-third of the papillary thyroid carcinoma (PTC) cases relapses, which directly impact on the quality of patients' lives. The molecular predictors of recurrence of PTC are poorly defined. We aimed at evaluating the long-term (10–20 years) prognostic value of aggressiveness markers in advanced PTC. To this end, immunohistochemistry for BRAF^V600E^, Estrogen receptor α, Progesterone receptor, Ki-67, and E-cadherin were performed in 53 primary advanced PTC from an up to 20 years follow-up patients from a well-characterized Brazilian cohort. Categorical data were summarized using frequencies and groups were compared using Chi-squared and Fisher's exact tests. The expressions of the aggressiveness markers were associated with clinical-pathological data using the single-covariate logistic regression analysis. The Kaplan-Meier method with the Log-rank and Peto tests was used to estimate the probability of PTC-free survival. Persistence and recurrence (active disease) were associated with age (≥55 years), tumor size (>2 cm), extrathyroidal extension, local aggressiveness, macroscopic lymph node metastasis, and TNM stage at initial treatment. The BRAF^V600E^ mutation status was associated with extrathyroidal extension, local aggressiveness, and inversely associated with distant metastasis at initial treatment. All progesterone receptor-positive patients had active disease and displayed a shorter time of PTC-free survival than the negative ones using the Kaplan-Meir analysis (*p* = 0.001, Log Rank; *p* = 0.005, Peto). Loss of E-cadherin expression was associated with an increase in the probability of active disease (OR = 3.75). BRAF^V600E^ could be useful as a biomarker of local aggressiveness, while PR positive and E-cadherin loss of expression could predict the recurrence of advanced PTC.

## Introduction

Thyroid carcinoma is the most frequent malignancy of the endocrine system (1) and the papillary thyroid carcinomas (PTC) account for about 80–85% of the cases (2). The vast majority of PTC has a good prognosis with a 5-year survival rate of 97% (3) although 1/3 of all cases persist or relapse (4), which directly impacts on the quality of patients' lives (5). Moreover, Tumor Node Metastasis (TNM) staging system fails to effectively predict the outcome of low-risk PTC patients at initial treatment (6). Thus, the identification of molecular markers that might help to predict patients' outcomes is clinically important for an effective initial approach.

Alterations in the mitogen-activated protein kinase (MAPK) pathway have been detected in 74.6% of PTC (7). Among them, the valine to glutamic acid exchange at amino acid 600 of BRAF (BRAF^V600E^) is the most prevalent mutation (around 58.46%) (7) and it exerts pleiotropic role in tumor cells, such as proliferation (8), epithelial to mesenchymal transition (EMT) (9), and loss of differentiation (10). The BRAF^V600E^ status has been correlated with several markers of metastasis and aggressiveness in different types of tumors, including PTC. Despite being extensively associated with local aggressiveness of PTC, the correlation between BRAF^V600E^ and persistence and recurrence is still contradictory (11). Fugazzola et al. (12) found that BRAF^V600E^ mutation status was not associated with recurrence or lethality in a cohort of 260 PTC patients with 6-years of follow-up. On the other hand, Elisei et al. (13) demonstrated a poor prognosis association of BRAF^V600E^ in a cohort of 319 low-risk patients with 5-years of follow-up. Above all, the question of whether BRAF^V600E^ could be a good predictor of distant metastasis of PTC patients remains controversial. Sancisi et al. (14) reported that BRAF^V600E^ is not related to distant metastasis in 47 cases of the distantly metastatic PTC with 9-years of follow-up while Xing et al. (15) proposed BRAF^V600E^ as a predictor of poor prognosis and distant metastasis in a cohort of 1,849 PTC with 3-years of follow-up.

The estrogen receptor α (ERα) is a nuclear receptor, responsible for estrogen-mediated genomic transcriptional effects in the nucleus or its interaction with other receptors, such as the epidermal growth factor receptor, outside nucleus compartment (16). The overexpression of ERα is associated with distant metastasis of PTC patients (17). Progesterone nuclear receptor (PR) is overexpressed in PTC in comparison with normal thyroid (18) and it is important to thyroid differentiation in the presence of thyrotropin (19). The concomitant positive PTC cases for BRAF^V600E^, ERα, and PR display local aggressiveness, increased tumor size without any impact on patients' overall survival (20). The Ki-67 proliferation index, an S phase antigen of the cell cycle, is higher in BRAF^V600E^ –positive PTC cases (21) and it is considered a predictor of lymph node dissemination, distant metastasis, and a worse prognosis for PTC patients (22). Moreover, BRAF^V600E^ reduces E-cadherin levels and increases the metastatic potential of thyroid carcinoma cell lines (9). E-cadherin is a cell adhesion molecule and its functional loss is associated with metastasis and worse prognosis in differentiated thyroid carcinomas (23).

The recent approval of BRAF inhibitor for patients with melanoma in Brazil and its promising results as a molecular target therapy for metastatic PTC patients (24) raised the question of whether this mutation has an impact on the clinical outcome of Brazilian patients with PTC, especially those with metastatic disease. Therefore, to evaluate the long-term (10–20 years) prognostic value of BRAF^V600E^ and its association with other PTC aggressiveness markers (ERα, PR, Ki-67, and E-cadherin), we studied a subpopulation of 53 patients from a well-characterized Brazilian cohort of 190 advanced PTC patients (25). To our knowledge, only one group reported the prognostic significance of BRAF^V600E^ in a Brazilian cohort (26), however, its association with the other prognostic markers has not been evaluated so far.

## Materials and Methods

### Ethics

This study was approved by the INCA HC-1 Ethics and Research Committees, protocol no. 86/2010, and was conducted following the principles of the Declaration of Helsinki and the Good Clinical Practice Guidelines.

### Sample Participants, Outcome Measures, and Exploratory Variables

We included 53 PTC patients with well-preserved tumor tissue samples available from a hospital-based cohort of 190 PTC patients (25). If we had calculated the sample size considering 50% of prevalence, 10% level of significance, 10% of error and a finite population of 190 patients, the number would be 50 patients (64 patients if 5% significance level). The eligible patients were aged 18 years or more and had undergone initial thyroid surgery to treat PTC between January 1st, 1990, and December 31st, 1999. The entry point for the beginning of the follow-up was the day of the initial surgical treatment. The hospital setting was a national cancer care referral center, affiliated with the Brazilian National Cancer Institute (INCA) in Rio de Janeiro (Brazil) that represents the majority of thyroid cancer cases (66%) attended in Rio de Janeiro during 2001–2009, according to the Hospital-based registries (RHC).

The primary outcomes were persistence/recurrence and PTC-free for morbidity. We considered only the event first experienced by the patient. Thus, patients who had persistent PTC were not considered at risk of recurrence. PTC persistence was defined as an evident residual disease (active disease status) until 12 months after initial surgical treatment. Furthermore, PTC recurrence was defined as having the first event of active disease occurring between 1 and 10 years of follow-up. Patients were considered PTC-free if they did not show active disease after the initial surgery with a minimum 1-year up to 10 years of follow-up. Active PTC disease was indicated when one or more of the following was observed: (a) structural disease evidenced by positive imaging findings or after radioactive iodine (I-131, RAI) therapy; and (b) biochemical evidence of disease with significant increase in serum thyroglobulin (Tg) levels, during thyroid hormone treatment (levothyroxine, LT4), over time compared with previously stable levels and/or an increase in serum Tg levels after LT4 withdrawal (stimulated Tg).

### Data Sources and Data Collection

The main sources for data collection were the medical charts of the hospital study setting and the national death registry. Consent has been obtained from each patient or subject after a full explanation of the purpose and nature of all procedures used. We used a structured questionnaire with open and closed questions. All surgical pathology reports were reviewed, and all positive macroscopic lymph nodes that were cN1 were also confirmed as positive in the pathology report (pN1).

### Immunohistochemistry (IHC)

BRAF^V600E^ mutation status, ERα, PR, and Ki-67 were evaluated using immunohistochemical analysis with specific antibodies (Supplementary Table 1) on a Ventana BenchMark Ultra® platform (Ventana, Tucson, AZ, USA), as previously described (27). Briefly, 4 μm cut sections from paraffin-embedded blocks were deparaffinized, washed with EZ Prep solution, pretreated with cell conditioner CC1 (pH 8) and endogenous peroxidase activity was blocked with H_2_O_2_. The Ventana staining procedure included incubation with the specific antibodies mentioned above, standard signal amplification with ultraWash, followed by chromogenic detection using ultraView Universal DAB detection kit and counterstained with Harris' hematoxylin. After that, slides were washed, dehydrated and mounted.

IHC for E-cadherin was performed on 4 μm cut paraffin sections of 51 PTC mounted on glass slides. Tissue sections of breast, obtained from the Department of Pathology of the Fluminense Federal University, served as the positive control. For antigen retrieval, the slides were incubated in a pH 6.0 solution (target antigen retrieval solution) for 45 min in a water bath at 96°C followed by a washing step with phosphate-buffered saline (PBS) and endogenous peroxidase activity was blocked with H_2_O_2_. Incubations with the primary antibody against E-cadherin (Supplementary Table 1) were performed overnight at 4°C. The E-cadherin antibody was incubated with biotinylated secondary antibodies using the streptavidin-biotin-peroxidase kit (Strep ABC complex/HRP Duet kit, DAKOCytomation). The reactions were developed with a solution containing diaminobenzidine tetrahydrochloride chromogen (DAB), and the sections were counterstained with Harris' hematoxylin. Negative and positive controls were included in all of the reactions.

All of the sections were reviewed independently by two pathologists (L.A.B and C.S.N) using an Olympus CX41 microscope (Olympus, Tokyo, Japan), who met to resolve discordant interpretations and establish a consensus categorization. For BRAF^V600E^, immunoreaction was scored positive when >10% of tumor cells exhibited diffuse cytoplasmic staining (27). Of note, all BRAF^V600E^ positive cases were stained for >50% of tumor cells. For ERα and PR, the classification used to score the IHC was the quickscore H-score ≥ 3 (28), which takes into account the product between the percentage of nuclear staining (1: 0–4%; 2: 5–19%; 3: 20–39%; 4: 40–59%; 5: 60–79%; 6: 80–100%) and the intensity (1: weak; 2: moderate; 3: strong) of positive cells. For Ki-67, a proliferation index was calculated for each tumor lesion by counting the total number of tumor cell nuclei and the Ki-67-positive nuclear cells (up to 100 cells) in randomly selected hotspot fields and then, the results were grouped in low (≤5% of positive cells), moderate (>5 and ≤10% of positive cells), and high risk (>10 and ≤30% of positive cells) (29). For E-cadherin, the positive slides were evaluated semi-quantitatively by the distribution of the immunohistochemical positivity of neoplastic cells. Whenever the distribution was 0% of stained cells, the cases were classified as negative, ≤50% as low expression and >50% as high expression.

### Statistical Analysis

Statistical analysis was performed using the R software package version 3.4.3 (www.R-project.org) and the SPSS (Statistical Package for the Social Sciences) version 20. Categorical data were summarized using absolute and relative frequencies. To compare groups, the Chi-squared (χ^2^) test or, for small cell sizes, Fisher's exact test was performed for categorical variables. Besides, to evaluate the demographic and clinicopathological factors to the biomarkers, the single- covariate logistic regression analysis was performed. The Kaplan-Meier (KM) method with the Log-rank and Peto tests was used to analyze the influence of BRAF^V600E^ mutation status, ERα, PR, Ki-67, and E-cadherin on the median time and the estimated probability of PTC-free survival. The American Statistical Association has highlighted an increasing critical of exclusive use of *p*-values to interpret the results (30). Thus, we have included 95% confidence intervals to estimate uncertainty and considered odds ratio between groups to estimate the effect size. We have added *p*-values as complementary information. Tests were two-sided using *P* < 0.05 to indicate statistically significant differences.

## Results

### Characterization of the Study Population

The study population (*n* = 53) was predominantly composed of females, the female-male ratio was 4:1, with a mean age of 44 years old (ranging from 18 to 81 years old). Tumors larger than 2 cm, locally aggressive (pT3+pT4) and with extrathyroidal extension were predominant. The presence of vessel invasion, macroscopic lymph node dissemination, and distant metastasis were frequent at initial treatment. As far as morbidity outcome is concerned, half of the cases had persistent or recurrent disease after a median follow-up of 13 years (ranging from 10 to 20 years). For statistical purposes, persistence and recurrence were grouped. According to histopathological classification, classic PTC were predominant (Supplementary Table 2). Regarding lethality, 8/53 cases died within 10 years of follow-up, according to the cancer death registry of the hospital. Of those, 6 cases died as a result of PTC. All patients' data are summarized in Table 1. For treatment, most patients underwent surgical treatment of total (73%) or near-total thyroidectomy (15%), lymph node dissection of the central compartment during initial surgery (44%) and RAI (70%) at INCA (96%).

**Table 1:** Single-covariate logistic regression of social demographic and clinicopathological variables data and outcome of 53 PTC patients.

**Variables**	**Outcome groups**						***p*-value**
	**PTC-free**		**Persistent + Recurrent**		**OR**	**95% CI**	
	***n* = 23**	**%**	***n* = 30**	**%**			
**Sex (*****n*** **= 53)**							
Male	–	–	10	33			
Female	23	100	20	67			
**Age (*****n*** **= 53)**							
<55 years	19	83	13	43	1.00		0.004^*^
≥55 years	4	17	17	57	6.21	(1.70–22.74)	
**Tumor size (*****n*** **= 53)**							
≤2 cm	14	61	10	33	1.00		0.046^**^
>2 cm	9	39	20	67	3.11	(1.01–9.63)	
**Multicentricity (*****n*** **= 50)**							
No	12	55	17	61	1.00		0.661^**^
Yes	10	45	11	39	0.78	(0.25–2.41)	
**Extrathyroidal extension (*****n*** **= 50)**							
No	14	64	6	21	1.00		0.002^**^
Yes	8	36	22	79	6.42	(1.83–22.46)	
**Vessel invasion (*****n*** **= 46)**							
No	12	67	11	39	3.09	(0.89–10.67)	0.070^*^
Yes	6	33	17	61			
**Local aggressiveness (*****n*** **= 51)**							
Locally limited (pT1+pT2)	15	68	9	30	1.00		0.006^**^
Locally aggressive (pT3+pT4)	7	32	21	70	5.00	(1.52–16.42)	
**Macroscopic lymph node metastasis** ***(pN1 or clinical, cN1)*** **(*****n*** **= 53)**							
No	15	65	8	27	1.00		0.005^*^
Yes	8	35	22	73	5.16	(1.58–16.77)	
**TNM stage at initial treatment (*****n*** **= 46)**							
Not advanced (I–II (≥45 years (base)	14	88	11	37	1.00		0.001^*^
Advanced (III–IV; II <45 years)	2	12	19	63	12.09	(2.30–6.42)	
**Distant metastasis at initial treatment (*****n*** **= 53)**							
Yes	–	–	9	30			
No	23	100	21	70			

PTC, papillary thyroid carcinoma; OR, odds ratio; CI, confidence interval;

* χ2;

** *Fisher's exact test; p < 0.05 was considered significant (in bold)*.

### Clinicopathological Variables Association With Persistence and Recurrence of PTC Patients

Concerning PTC patients' morbidity outcome, most of the clinicopathological variables, larger tumors (>2 cm) and the presence of extrathyroidal extension, local aggressiveness, macroscopic lymph node, and distant metastasis, as well as an advanced disease at initial treatment, were associated with persistence and recurrence. Moreover, social and demographic data analysis showed that age ≥55 years increased the probability of persistence and recurrence disease. All the details about the association of social/demographic and clinicopathological data with patients' morbidity outcomes are summarized in Table 1.

### Progesterone Receptor Expression and Loss of E-cadherin Are Associated With Persistence and Recurrence of PTC Patients

**Figure 1:**
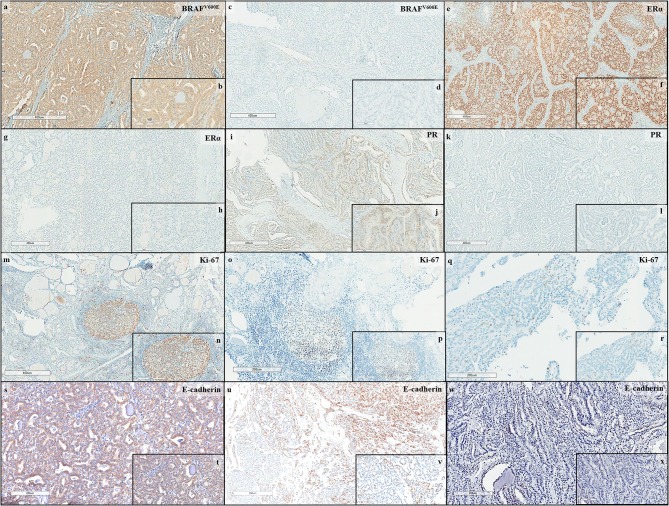
Immunohistochemical expression in PTC: BRAF^V600E^ **(a,b)** Positive immunostaining (*5X, 20X*) and **(c,d)** Negative immunostaining (*5X, 20X*); Estrogen Receptor α (ERα) **(e,f)** Positive immunostaining (*5X, 20X*) and **(g,h)** Negative immunostaining (*5X, 20X*); Progesterone Receptor (PR) **(i,j)** Positive immunostaining (*5X, 20X*); **(k,l)** Negative immunostaining (*5X, 20X*); Ki-67 **(m,n)** High risk immunostaining (*5X, 10X*), **(o,p)** Moderate risk immunostaining (*10X, 20X*), and **(q,r)** Low risk immunostaining (*10X, 20X*); and E-cadherin **(s,t)** High immunostaining (*10X, 20X*), **(u,v)** Low immunostaining (*4X, 20X*), and **(w,x)** Negative immunostaining (*10X, 20X*).

The BRAF^V600E^, ERα, PR, Ki-67, and E-cadherin protein expressions were assessed by immunohistochemical staining and the representative figures of immunostaining for the five targets are illustrated in Figure 1. In our study population, 24/53 (45.2%), 19/53 (35.8%), and 10/48 (20.8%) PTC cases were positive for the BRAF^V600E^ mutation, ERα, and PR, respectively. Moreover, 14/49 cases (28.5%) were classified as moderate to high-risk PTC, according to Ki-67 index levels. Apart from that, 37/51 PTC cases (72.5%) displayed a negative and low expression of E-cadherin.

**Figure 2:**
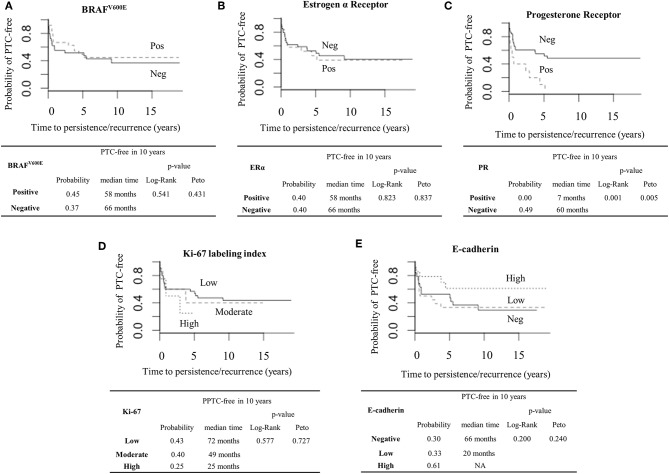
Prognostic values of BRAF^V600E^, Estrogen Receptor α (ERα), Progesterone Receptor (PR), Ki-67, and E-cadherin in PTC patients. Kaplan-Meier curve with Log Rank and Peto tests were used to estimate the probability and the median time of PTC-free with the median of 10 years of follow-up, using the following molecular biomarkers: **(A)** BRAF^V600E^ (Positive/Negative); **(B)** ERα (Positive/Negative); **(C)** PR (Positive/Negative); **(D)** Ki-67 (Low, Moderate, and High risk); **(E)** E-cadherin (Negative, Low, and High expression).

The impact of BRAF^V600E^, ERα, PR, Ki-67, and E-cadherin protein expressions on the persistence and recurrence of PTC patients was analyzed (Table 2). All PR-positive PTC patients had persistence or recurrence. The PR-positive patients displayed a shorter time of PTC-free survival than PR-negative ones as shown by the Kaplan-Meir method (*p* = 0.001, Log Rank; *p* = 0.005, Peto), with the probability of PTC-free in 10 years of 0.0 (95% CI = NA–NA) for the PR-positive and 0.49 (95% CI = 0.347–0.983) for the PR-negative, as shown in Figure 2. In agreement with that, the median time of persistence and recurrence was about 7 months for the PR-positive and 6 years in the PR-negative patients. No significant influence on PTC-free survival time for the other molecular biomarkers tested was detected (Figure 2). Absence and decreased expression of E-cadherin were associated with an increase in the probability of active disease (OR = 3.75; 95% CI = 1.03–13.65) (Table 2). Of note, the presence of BRAF^V600E^ mutation was associated with an increased chance of extrathyroidal extension (OR = 3.50; 95% CI = 1.05–11.66) and local aggressiveness (OR = 3.24; 95% CI = 1.02–10.28) while it decreased the probability of having distant metastasis (OR = 0.11; 95% CI = 0.01–0.99) at initial treatment (Supplementary Table 3).

**Table 2:** Single-covariate logistic regression of BRAF^V600E^, ER-α, PR, Ki-67, and E-cadherin protein expression with morbidity outcome of PTC patients.

**Molecular biomarkers**		**Outcome groups**		**OR**	**95% CI**	***p*-value**
		**PTC-free**	**Persistence + Recurrence**			
BRAF^V600E^ (*n* = 53)	Neg (*n* = 29)	12 (52%)	17 (57%)	1.00	(0.28–2.48)	0.745^*^
	Pos (*n* = 24)	11 (48%)	13 (43%)	0.83		
ER-α (*n* = 53)	Neg (*n* = 34)	15 (65%)	19 (63%)	1.00	(0.35–3.38)	0.887^*^
	Pos (*n* = 19)	8 (35%)	11 (37%)	1.09		
PR (*n* = 48)	Neg (*n* = 38)	19 (100%)	19 (65%)			
	Pos (*n* = 10)	–	10 (35%)			
Ki67 (*n* = 49)	Low (*n* = 35)	16 (76%)	19 (68%)	1.00	(0.42–5.45)	0.524^**^
	Moderate (*n* = 10)	4 (19%)	6 (21%)	1.52		
	High (*n* = 4)	1 (5%)	3 (11%)			
E-cadherin (*n* = 51)	High (*n* = 14)	9 (43%)	5 (17%)	1.00	(1.03–13.65)	**0.045**^#^
	Low (*n* = 18)	6 (29%)	12 (40%)	3.75		
	Neg (*n* = 19)	6 (29%)	13 (43%)			

PTC, papillary thyroid carcinoma; BRAF^V600E^, valine (V) to a glutamic acid (E) amino acid substitution at position 600 in BRAF; ER-α, Estrogen receptor α; PR, Progesterone receptor; OR, Odds ratio; CI, confidence interval;

# negative + low expression vs. High expression of E-cadherin;

* χ^2^;

** *Fisher's exact test; p < 0.05 was considered significant (in bold)*.

Taken together, our data indicate that the presence of PR and the reduced expression of E-cadherin could confer worse prognosis of PTC patients, no matter in the presence or absence of BRAF^V600E^. It is noteworthy that no statistically significant association of BRAF^V600E^ mutation status, ERα, PR, Ki-67, and E-cadherin protein expressions with one another was found in our study population (data not shown).

## Discussion

The advantages of our study are: (1) the long-term follow-up (median 13 years); (2) relative higher prevalence of active disease (persistence and recurrence) than previous studies (1.4–26%) (31–33), and the investigation of the potential prognostic value of five molecular biomarkers (BRAF^V600E^, ERα, PR, Ki-67, and E-cadherin). On the other hand, the main limitation is the relatively small sample and the low number of events *per* variable that reduces the power of the study but it still representative of a well-characterized cohort of 190 PTC patients (25). Indeed, our study is descriptive. Moreover, no differences in age, TNM staging and active disease were observed between the larger cohort (*n* = 190) and our convenience sample (*n* = 53). Of note, despite of our efforts to compare our findings with larger PTC datasets, such as PTC-TGGA database (7), the characteristics of the both populations were remarkably different: the majority of TCGA cases were locally limited (54%, pT1+pT2), distant metastasis rate was scarce (0.5%) and the frequency of active disease was lower (5.4%), restraining further validation.

Despite following an indolent course (4), our data revealed an aggressive behavior of PTC in the study population at initial treatment. Locally aggressive PTC and distant metastasis were up to six times more frequent than previous reports in Japan (31), Italy (32), and the USA (33). These findings might be due to the advanced disease (TNM III/IV), which increases the frequencies of micrometastasis and reduces the chances of total tumor resection (34), contributing to active disease after initial treatment, probably due to late diagnosis of PTC. Interestingly, 1/3 of the cases of active disease group were classified as not advanced PTC (TNM I/II), suggesting that the TNM system failed at predicting morbidity outcome for these cases. TNM accuracy to predict outcome varies with the type of tumor (35). Thus, the search for molecular biomarkers is important to allow an early identification of patients who will probably evolve with a more aggressive disease.

In this context, our data corroborate with previous studies (13–15), in which BRAF^V600E^ was associated with local aggressiveness of PTC. Even though the presence of this mutation had no direct influence on persistence and recurrence, it was associated with high-risk clinicopathologic features of PTC that predict patients' outcomes. So, the prognostic value of BRAF^V600E^ cannot be excluded. Moreover, our data suggest that BRAF^V600E^ does not seem to increase the risk of distant metastasis in PTC, as previously described (14, 36). The authors have reported that the presence of BRAF^V600E^ was associated with local aggressiveness and lymph node dissemination but inversely correlated with distant metastasis, suggesting that BRAF^V600E^ positive clones might have a disadvantage of survival in the blood despite of being associated with invasiveness. As for ERα, no impact on aggressiveness or outcome was observed in our study population. The role of ERα on thyroid cancer is still controversial. While some published data observed no association (37) or a protective role of ERα on the thyroid cancer remission (38), others associated ERα-positive PTC with a more aggressive presentation (17). Since in these previous studies not advanced (TNM I) PTC cases were predominant, ERα might be important at initial but not in late steps of PTC progression. Recently, it was shown that the 36 kDa variant of full-length ERα (66 kDa), ERα36, was associated with the aggressive behavior of PTC by inhibiting full-length ERα-mediated genomic effects (37). Yet, the ERα antibody used in this study cannot recognize the ERα36 variant.

Regarding PR, its presence shortens the time of PTC-free survival during all the follow-up period of the patients and, therefore, our data suggest that PR could have prognostic value by predicting active disease. However, the mechanism by which PR contributes to PTC progression remains to be elucidated. Interestingly, progesterone potentiates thyrotropin-mediated transcription effects in the thyroid (19), which might be important to PTC progression. Recently, PR expression has been implicated in breast cancer cell dedifferentiation through miR-141/*STAT5a* pathway (39) and shorter relapse-free survival in breast cancer (40), suggesting that a dedifferentiation phenotype might contribute to an aggressive behavior of PR-positive tumors, which is agreement with our data.

Despite its biological relevance in proliferation (22), no statistically significant differences in tumor size and Ki-67 index levels were observed. These results could be due to the sample number and or/ the cut-off (≤ or >2 cm) applied. Indeed, when analyzed as a continuous variable, a direct proportion between Ki-67 index levels and tumor median size was found. As far as E-cadherin immunostaining is concerned, its loss is associated with distant metastasis in the outcome and active disease and thus, it could be useful to identify PTC patients with unfavorable clinical outcomes, as previously reported (23). One possible mechanism involved in the downregulation of E-cadherin is the hypermethylation of its promoter region, previously reported in 80% of PTC cases (41). No molecular alterations in patterns of E-cadherin (P- and N-cadherin) were found in PTC (42). It was shown that BRAF^V600E^ increases the ability of invasiveness in PTC cells by decreasing E-cadherin expression (9). However, we found no association between BRAF^V600E^ and loss of E-cadherin in our present PTC cohort, probably due to the small sample size. In fact, lack of statistical significance should not be considered the only criteria to determine whether there is no biological effect (30).

In conclusion, our data indicate that BRAFV600E might be important to confer initial local aggressiveness, while PR presence and E-cadherin loss of expression could predict persistence or recurrence of PTC. Together with the clinicopathological findings, these molecular biomarkers could help predict PTC patients' outcomes. Our study findings encourage the design of larger confirmatory studies.

## Data Availability Statement

The data generated for this study are available on request to the corresponding author.

## Ethics Statement

The studies involving human participants were reviewed and approved by INCA HC-1 Ethics and Research Committees. The patients/participants provided their written informed consent to participate in this study.

## Author Contributions

TC and RP were responsible for the integrity of the work as a whole, as well as performed and designed the research study, analyzed data, and wrote the paper. TC, RO, and FC performed the statistical analyses. LB performed the E-cadherin immunostaining and analyzed data. FA and LP reclassified all primary tumor samples and analyzed data. DC designed the research study, analyzed data, and revised the paper. All authors have contributed significantly and agree with the content of the manuscript.

### Conflict of Interest

The authors declare that the research was conducted in the absence of any commercial or financial relationships that could be construed as a potential conflict of interest.
