# Supplementary material for: Molecular Predictors for Advanced Papillary Thyroid Carcinoma Recurrence
Source: Front Endocrinol (Lausanne). 2019 Dec 5;10:839. doi: 10.3389/fendo.2019.00839 (PMC6907036; doi:10.3389/fendo.2019.00839)
Supplement: Supplementary file 1 [file Table_1.DOCX]

Supplementary Material

# Supplementary Tables

**Supplementary Table 1.** Antibodies and conditions used for immunohistochemistry in this study

| **Antibody** | **Clone/Specie** | **Manufacturer** | **Cell conditioner/ Antigen retrieval**  **(min/ solution)** | **Incubation/**  **Secundary Ac** | **Diluition** |
| --- | --- | --- | --- | --- | --- |
| **BRAF^V600E^** | VE1 /mouse monoclonal | Spring | 64 min/CC1 | 16 min/ Optiview | 1:100 |
| **ER-α** | SP1 /rabbit monoclonal | Ventana | 64min/CC1 | 16 min/  UltraView DAB | Pre-diluited |
| **PR** | 1E2 /rabbit monoclonal | Ventana | 64min/CC1 | 16 min/  UltraView DAB | Pre-diluited |
| **Ki-67** | SP6 / rabbit monoclonal | CellMarque | 64min/CC1 | 32 min | 1:300 |
| **E-cadherin** | NCH38/mouse monoclonal | DAKOCytomation | 45 min/pH 6.0 - Target antigen  Retrieval solution | 30 min/Strep ABC complex –  HRP Duet kit | 1:50 |

BRAF^V600E^= valine (V) to a glutamic acid (E) amino acid substitution at position 600 in BRAF; ER-α= Estrogen receptor α; PR= Progesterone receptor; DAB= 3, 3’ -diaminobenzidine

**Supplementary Table 2**. Prevalence of PTC variants (n=53)

|  |  |  |
| --- | --- | --- |
| PTC variants Prevalence | | |
| N=53 (100%) | | |
| **Classification** | **n** | **%** |
| Classic | 42 | 79 |
| Follicular | 5 | 9 |
| Tall cells | 2 | 4 |
| Clear cell | 1 | 2 |
| Solid | 1 | 2 |
| Oncocytic | 1 | 2 |
| Whartin-like | 1 | 2 |

PTC= Papillary thyroid carcinoma

**Supplementary Table 3.** Single-covariate logistc regression of BRAF^V600E^, ER-α, PR, Ki-67 and E-cadherin protein expression with social and demographic and clinicopathological features of PTC patients

|  |  | |  | |  | |  | |  | |  | |  |  |  |  | |  |  | |  | |  | |  |  | |  | |  | |  | |  | |  | |  | |  | |  | |  |  | |  |
| --- | --- | --- | --- | --- | --- | --- | --- | --- | --- | --- | --- | --- | --- | --- | --- | --- | --- | --- | --- | --- | --- | --- | --- | --- | --- | --- | --- | --- | --- | --- | --- | --- | --- | --- | --- | --- | --- | --- | --- | --- | --- | --- | --- | --- | --- | --- | --- |
| **Molecular Biomarkers** | **Molecular Biomarkers** | | | | | | | | | | | | | | | | | | | | | | | | | | | | | | | | | | | | | | | | | | | | | | |
|  | **BRAF^V600E^ (n=53)** | | | | | | **ER-α (n=53)** | | | | | | **PR (n=48)** | | | | | | | | | **Ki67 (n=49)** | | | | | | | | | | | | | **E-cadherin (n=51)** | | | | | | | | | | | | |
| ***χ2; **Fisher's exact test** | **Neg (n=29)** | **Pos (n=24)** | **OR** | **95% CI** | | **p- value** | **Neg (n=34)** | **Pos (n=19)** | | **OR** | **95% CI** | **p-value** | **Neg (n=38)** | **Pos (n=10)** | **OR** | | **95% CI** | | | **p-value** | | **Low (n=35)** | | **Moderate (n=10)** | | | **High (n=4)** | | **OR^#^** | | **95% CI** | | **p-value** | | **Neg (n=19)** | | **Low (n=18)** | | **High (n=14)** | | **OR^$^** | | **95% CI** | | | **p-value** | |
| **Sex (n=53)** |  |  |  |  | |  |  |  | |  |  |  |  |  |  | |  | | |  | |  | |  | | |  | |  | |  | |  | |  | |  | |  | |  | |  | | |  | |
| Male | 7(24%) | 3(12%) | 1.00 |  | | 0.318* | 6(18%) | 4(21%) | | 1.00 |  | 0.761** | 5(13%) | 4(40%) | 1.00 | |  | | | 0.075** | | 8(23%) | | 1(10%) | | | 1(25%) | | 1.00 | |  | | 0.724** | | 5(26%) | | 2(11%) | | 3(22%) | | 1.00 | |  | | | 0.840* | |
| Female | 22(76%) | 21(88%) | 2.23 | (0.51-9.77) | |  | 28(82%) | 15(79%) | | 0.80 | (0.20-3.30) |  | 33(87%) | 6(60%) | 0.23 | | (0.05-1.10) | | |  |  | 27(77%) | | 9(90%) | | | 3(75%) | | 0.56 | | (0.10-3.06) | |  |  | 14(74%) | | 16(89%) | | 11(78%) | | 0.86 | | (0.19-3.91) | | |  |  |
| **Age (n=53)** |  |  |  |  | |  |  |  | |  |  |  |  |  |  | |  | | |  | |  | |  | | |  | |  | |  | |  | |  | |  | |  | |  | |  | | |  | |
| <55 years | 18(62%) | 14(58%) | 1.00 |  | | 0.782* | 19(56%) | 13(68%) | | 1.00 |  | 0.371* | 25(66%) | 5(50%) | 1.00 | |  | | | 0.468** | | 22(63%) | | 6(60%) | | | 1(25%) | | 1.00 | |  | | 0.408** | | 13(68%) | | 10(56%) | | 7(50%) | | 1.00 | |  | | | 0.433* | |
| ≥55 years | 11(38%) | 10(42%) | 1.17 | (0.39-3.53) | |  | 15(44%) | 6(32%) | | 0.58 | (0.18-1.90) |  | 13(34%) | 5(50%) | 1.92 | | (0.47-7.87) | | |  |  | 13(37%) | | 4(40%) | | | 3(75%) | | 1.69 | | (0.48-5.92) | |  |  | 6(32%) | | 8(44%) | | 7(50%) | | 1.64 | | (0.47-5.68) | | |  |  |
| **Tumor size (n=53)** |  |  |  |  | |  |  |  | |  |  |  |  |  |  | |  | | |  | |  | |  | | |  | |  | |  | |  | |  | |  | |  | |  | |  | | |  | |
| ≤2 cm | 15(52%) | 9(38%) | 1.00 |  | | 0.302* | 16(47%) | 8(42%) | | 1.00 |  | 0.728* | 17(45%) | 3(30%) | 1.00 | |  | | | 0.488** | | 18(51%) | | 2(20%) | | | 1(25%) | | 1.00 | |  | | 0.065* | | 8(42%) | | 8(44%) | | 6(43%) | | 1.00 | |  | | | 0.980* | |
| >2 cm | 14(48%) | 15(62%) | 1.79 | (0.59-5.37) | |  | 18(53%) | 11(58%) | | 1.22 | (0.39-3.79) |  | 21(55%) | 7(70%) | 1.89 | | (0.42-8.43) | | |  |  | 17(49%) | | 8(80%) | | | 3(75%) | | 3.88 | | (0.92-16.36) | |  |  | 11(58%) | | 10(56%) | | 8(57%) | | 1.02 | | (0.29-3.52) | | |  |  |
| **Multicentricity**  **(n=50)** |  |  |  |  | |  |  |  | |  |  |  |  |  |  | |  | | |  | |  | |  | | |  | |  | |  | |  | |  | |  | |  | |  | |  | | |  | |
| No | 16(60%) | 12(52%) | 1.00 |  | | 0.987* | 16(52%) | 13(68%) | | 1.00 |  | 0.382* | 18(52%) | 8(80%) | 1.00 | |  | | | 0.154** | | 19(60%) | | 4(40%) | | | 2(50%) | | 1.00 | |  | | 0.640** | | 8(44%) | | 9(65%) | | 9(64%) | | 1.00 | |  | | | 0.524* | |
| Yes | 11(40%) | 11(48%) | 1.56 | (0.50-4.83) | |  | 15(48%) | 6(32%) | | 0.49 | (0.15-1.63) |  | 17(48%) | 2(20%) | 0.26 | | (0.05-1.43) | | |  |  | 13(40%) | | 6(60%) | | | 2(50%) | | 1.95 | | (0.55-6.95) | |  |  | 10(56%) | | 6(35%) | | 5(36%) | | 0.66 | | (0.18-2.37) | | |  |  |
| **Extrathyroidal extension (n=50)** |  |  |  |  | |  |  |  | |  |  |  |  |  |  | |  | | |  | |  | |  | | |  | |  | |  | |  | |  | |  | |  | |  | |  | | |  | |
| No | 14(54%) | 6(25%) | 1.00 |  | | **0.025*** | 12(38%) | 8(44%) | | 1.00 |  | 0.857* | 15(40%) | 2(22%) | 1.00 | |  | | | 0.450** | | 14(42%) | | 1(11%) | | | 2(50%) | | 1.00 | |  | | 0.229** | | 7(41%) | | 6(33%) | | 5(39%) | | 1.00 | |  | | | 0.933* | |
| Yes | 12(46%) | 18(75%) | 3.50 | (1.05-11.66) | |  | 20(62%) | 10(56%) | | 0.75 | (0.23-2.42) |  | 22(60%) | 7(78%) | 2.39 | | (0.44-13.10) | | |  |  | 19(58%) | | 8(89%) | | | 2(50%) | | 2.46 | | (0.57-10.61) | |  |  | 10(59%) | | 12(67%) | | 8(61%) | | 0.94 | | (0.26-3.51) | | |  |  |
| **Vessel invasion (n=46)** |  |  |  |  | |  |  |  | |  |  |  |  |  |  | |  | | |  | |  | |  | | |  | |  | |  | |  | |  | |  | |  | |  | |  | | |  | |
| No | 14(56%) | 10(48%) | 1.00 |  | | 0.778* | 12(43%) | 11(61%) | | 1.00 |  | 0.365* | 15(47%) | 5(50%) | 1.00 | |  | | | 0.860** | | 15(52%) | | 2(22%) | | | 3(75%) | | 1.00 | |  | | 0.428* | | 8(47%) | | 5(33%) | | 9(70%) | | 1.00 | |  | | | 0.090* | |
| Yes | 11(44%) | 11(52%) | 1.19 | (0.37-3.81) | |  | 16(57%) | 7(39%) | | 0.48 | (0.14-1.60) |  | 17(53%) | 5(50%) | 0.88 | | (0.21-3.65) | | |  |  | 14(48%) | | 7(78%) | | | 1(25%) | | 1.71 | | (0.45-6.51) | |  |  | 9(53%) | | 10(67%) | | 4(30%) | | 0.38 | | (0.08-1.20) | | |  |  |
| **Local aggressiveness (n=51)** |  |  |  |  | |  |  |  | |  |  |  |  |  |  | |  | | |  | |  | |  | | |  | |  | |  | |  | |  | |  | |  | |  | |  | | |  | |
| Locally limited (pT1+pT2) | 18(62%) | 7(30%) | 1.00 |  | | **0.029*** | 14(42%) | 10(53%) | | 1.00 |  | 0.672* | 16(43%) | 3(30%) | 1.00 | |  | | | 0.718** | | 18(52%) | | 3(30%) | | | 1(33%) | | 1.00 | |  | | 0.208** | | 11(58%) | | 4(22%) | | 7(54%) | | 1.00 | |  | | | 0.408* | |
| Locally aggressive (pT3 + pT4) | 11(38%) | 16(70%) | 3.24 | (1.02-10.28) | |  | 19(58%) | 9(47%) | | 0.66 | (0.21-2.06) |  | 21(57%) | 7(70%) | 1.78 | | (0.40-7.97) | | |  |  | 17(48%) | | 7(70%) | | | 2(67%) | | 2.38 | | (0.62-9.20) | |  |  | 8(42%) | | 14(78%) | | 6(46%) | | 0.58 | | (0.16-2.09) | | |  |  |
| **Macroscopic lymph node metastasis *(pN1 or clinical, cN1*(n=53)** |  |  |  |  | |  |  |  | |  |  |  |  |  |  | |  | | |  | |  | |  | | |  | |  | |  | |  | |  | |  | |  | |  | |  | | |  | |
| No | 13(50%) | 9(53%) | 1.00 |  | | 1.000** | 17(57%) | 6(46%) | | 1.00 |  | 0.763* | 18(60%) | 3(33%) | 1.00 | |  | | | 0.255** | | 15(52%) | | 3(43%) | | | 3(75%) | | 1.00 | |  | | 0.207** | | 9(50%) | | 5(39%) | | 8(73%) | | 1.00 | |  | | | 0.160* | |
| Yes | 13(50%) | 8(47%) | 1.56 | (0.52-4.68) | |  | 13(43%) | 7(54%) | | 1.52 | (0.48-4.81) |  | 12(40%) | 6(67%) | 2.10 | | (0.47-9.36) | | |  |  | 14(48%) | | 4(57%) | | | 1(25%) | | 2.36 | | (0.62-8.98) | |  |  | 9(50%) | | 8(61%) | | 3(27%) | | 0.41 | | (0.12-1.43) | | |  |  |
| **TNM stage at initial treatment (n=46)** |  |  |  |  | |  |  |  | |  |  |  |  |  |  | |  | | |  | |  | |  | | |  | |  | |  | |  | |  | |  | |  | |  | |  | | |  | |
| *Not advanced* (I-II (≥ 45 yrs (base) | 14(56%) | 13(62%) | 1.00 |  | | 0.387* | 17(57%) | 8(50%) | | 1.00 |  | 0.666* | 17(53%) | 5(50%) | 1.00 | |  | | | 0.860** | | 17(59%) | | 5(50%) | | | 1(25%) | | 1.00 | |  | |  | | 10(59%) | | 7(41%) | | 6(60%) | | 1.00 | |  | | | 0.579* | |
| *Advanced* (III-IV; II <45 yrs | 11(44%) | 8(38%) | 0.57 | (0.18-1.85) | |  | 13(43%) | 8(50%) | | 1.31 | (0.39-4.42) |  | 15(47%) | 5(50%) | 1.13 | | (0.27-4.69) | | |  |  | 12(41%) | | 5(50%) | | | 3(75%) | | 1.89 | | (0.52-6.87) | | 0.334** | | 7(41%) | | 10(59%) | | 4(40%) | | 0.67 | | (0.16-2.79) | | |  |  |
| **Distant metastasis at initial treatment (n=53)** |  |  |  |  | |  |  |  | |  |  |  |  |  |  | |  | | |  | |  | |  | | |  | |  | |  | |  | |  | |  | |  | |  | |  | | |  | |
| No | 21(72%) | 23(96%) | 1.00 |  | | **0.031*** | 28(82%) | 16(84%) | | 1.00 |  | 1.000** | 32(84%) | 7(70%) | 1.00 | |  | | | 0.369** | | 28(80%) | | 9(90%) | | | 4(100%) | | 1.00 | |  | | 0.293** | | 16(84%) | | 14(78%) | | 12(86%) | | 1.00 | |  | | | 0.699* | |
| Yes | 8(28%) | 1(4%) | 0.11 | (0.01-0.99) | |  | 6(18%) | 3(16%) | | 0.88 | (0.19-3.98) |  | 6(16%) | 3(30%) | 2.29 | | (0.46-11.43) | | |  |  | 7(20%) | | 1(10%0 | | | 0(0%) | | 0.31 | | (0.03-2.77) | |  |  | 3(16%) | | 4(22%) | | 2(14%) | | 0.72 | | (0.13-3.94) | | |  |  |
|  |  |  |  |  | |  |  |  | |  |  |  |  |  |  | |  | | |  | |  | |  | | |  | |  | |  | |  | |  | |  | |  | |  | |  | | |  | |

PTC= papillary thyroid carcinoma; BRAF^V600E^= valine (V) to a glutamic acid (E) amino acid substitution at position 600 in BRAF; ER-α= Estrogen receptor α; PR= Progesterone receptor; OR= odds ratio; CI= confidence interval; *χ2; **Fisher's exact test; P < 0.05 was considered significant
